# JC Polyomavirus-Associated Nephropathy Case Report: Clinical and Laboratory Learning

**DOI:** 10.3389/bjbs.2025.14170

**Published:** 2025-03-28

**Authors:** Rachael M. Tomb, Siobhan K. McManus, David Kipgen, Sawsan Yaqub, Sally Taylor, Rory N. Gunson

**Affiliations:** ^1^ West of Scotland Specialist Virology Centre, Glasgow Royal Infirmary, Glasgow, United Kingdom; ^2^ Glasgow Renal and Transplant Unit, Queen Elizabeth University Hospital, Glasgow, United Kingdom; ^3^ Pathology Department, Queen Elizabeth University Hospital, Glasgow, United Kingdom

**Keywords:** JC virus, John Cunningham virus, PVAN, polyomavirus nephropathy, case report

## Abstract

**Introduction:**

John Cunningham (JC) virus is commonly associated with progressive multifocal leukoencephalopathy. However, this polyomavirus can also be a rare etiological agent of nephropathy in renal transplant recipients. Polyomavirus-associated nephropathy (PVAN) can be difficult to treat, resulting in graft dysfunction and failure.

**Details:**

We report a rare case of JC-PVAN in a deceased donor kidney transplant recipient. Following a decline in renal function approximately 4 years post-transplant, the patient underwent biopsy and SV40 staining. A diagnosis of early/mild PVAN was made. Confirmatory PCR testing for BK virus, the virus most commonly associated with PVAN, was repeatedly negative. PCR for JC virus, a much rarer cause of nephropathy, was not performed as testing was not within our laboratory testing scope. Approximately 6 years post-transplant, following further pathological examination and exclusion of BK virus, JC virus was confirmed as the cause of graft dysfunction via off-scope PCR testing. Reductions in immunosuppression were implemented following the initial PVAN diagnosis, however, decline in renal function continued. The patient returned to haemodialysis 8 years post-transplant.

**Discussion:**

This paper highlights the challenges faced achieving the diagnosis of JC virus and importance of collaboration between clinical and laboratory teams to ensure appropriate testing to aid diagnosis. In addition, we aim to increase the inclusion of JC virus in the differential diagnosis in cases of nephropathy in allograft recipients with unclear aetiology.

## Introduction

Two of the polyomaviruses associated with human infection are BK virus and John Cunningham (JC) virus [1]. These viruses are ubiquitous in the population and, following primary infection, can establish latency in the kidney uroepithelium [2]. However, upon intense immunosuppression, BK and JC virus can reactivate, causing infection [1, 2]. Within the kidney allograft recipient population, these viruses are associated with graft dysfunction termed “polyomavirus-associated nephropathy” (PVAN), which can eventually lead to graft loss [3]. The incidence of BK-PVAN is relatively high, developing in up to 15% of kidney transplant recipients in the first year post-transplant, resulting in 15%–50% graft loss [2, 4]. JC-PVAN is much rarer, occurring in 0.9%–3% of kidney transplant recipients either early or late post-transplant [1, 5].

Definitive diagnosis of PVAN requires demonstration of the virus in renal tissue, usually by staining with a SV40 immunohistochemistry stain to detect polyomavirus large T antigen [2, 6]. However, this method cannot differentiate between BK and JC virus [1]. Molecular detection is relied upon to type the polyomavirus detected during SV40 staining. Within our laboratory, the West of Scotland Specialist Virology Centre (WoSSVC), in cases of post-solid organ transplant nephropathy we would routinely test recipients for BK virus in plasma, as well as cytomegalovirus (CMV) and adenovirus using real-time PCR assays [7]. Owing to the rarity of JC-PVAN, testing for JC virus in cases of post-transplant nephropathy is not within our testing scope. Further to this, JC virus is generally not included as a target within commercially available testing kits used within NHS laboratories. This report demonstrates the challenging nature of diagnosing JC virus-associated nephropathy and highlights the importance of appropriate molecular laboratory testing when JC-PVAN is suspected.

## Case Presentation

This case involves a 41-year-old male with a three-and-a-half year history of dialysis-dependent renal failure secondary to chronic pyelonephritis, who underwent a first deceased donor kidney transplant with a 211 HLA-mismatched graft. The patient received the standard post-kidney transplant immunosuppression with basiliximab induction therapy and tacrolimus (to achieve levels 4–8 ng/mL), mycophenolate mofetil 1 g twice daily, and prednisolone tapered to 5 mg daily [8]. After a period of delayed graft function, during which the patient had pulsed methylprednisolone for suspected rejection, a biopsy showed histological evidence of acute tubular necrosis only. Four months post-transplant, an eGFR (CKD-EPI) of 50 mL/min/1.73 m^2^ was achieved. The patient sustained a myocardial infarction 2 years post-transplant that did not significantly impact on renal function.

Four years post-transplant, the patient’s creatinine rose due to suspected viral gastroenteritis and, as a result, mycophenolate mofetil was reduced to 750 mg twice daily. PCR testing at that time showed no evidence of BK infection. Additionally CMV was not detected using a multiplex PCR transplant screen [7]. The patient’s tacrolimus levels were variable, and dose adjustments were made in an effort to keep them within range. There was a subsequent progressive decline in eGFR to 30 mL/min/1.73 m^2^ over the following months, resulting in a biopsy being undertaken. Upon biopsy, there were no viral inclusions identified on H&E, however, there was positive SV40 large T antigen immunoperoxidase staining in tubular epithelial cell nuclei (1% of tubules) without surrounding interstitial inflammation. As there was positive SV40 staining and no evidence of rejection, a diagnosis of early/mild polyomavirus nephropathy was made. Two plasma samples taken 1 month apart, prior to and following biopsy, were both negative for BK PCR. JC PCR was requested following the biopsy findings but declined as the WoSSVC does not routinely test plasma for JC virus in cases of post-transplant nephropathy. The patient’s immunosuppression was reduced: mycophenolate mofetil was reduced to 500 mg twice daily and tacrolimus dose was reduced.

**FIGURE 1:**
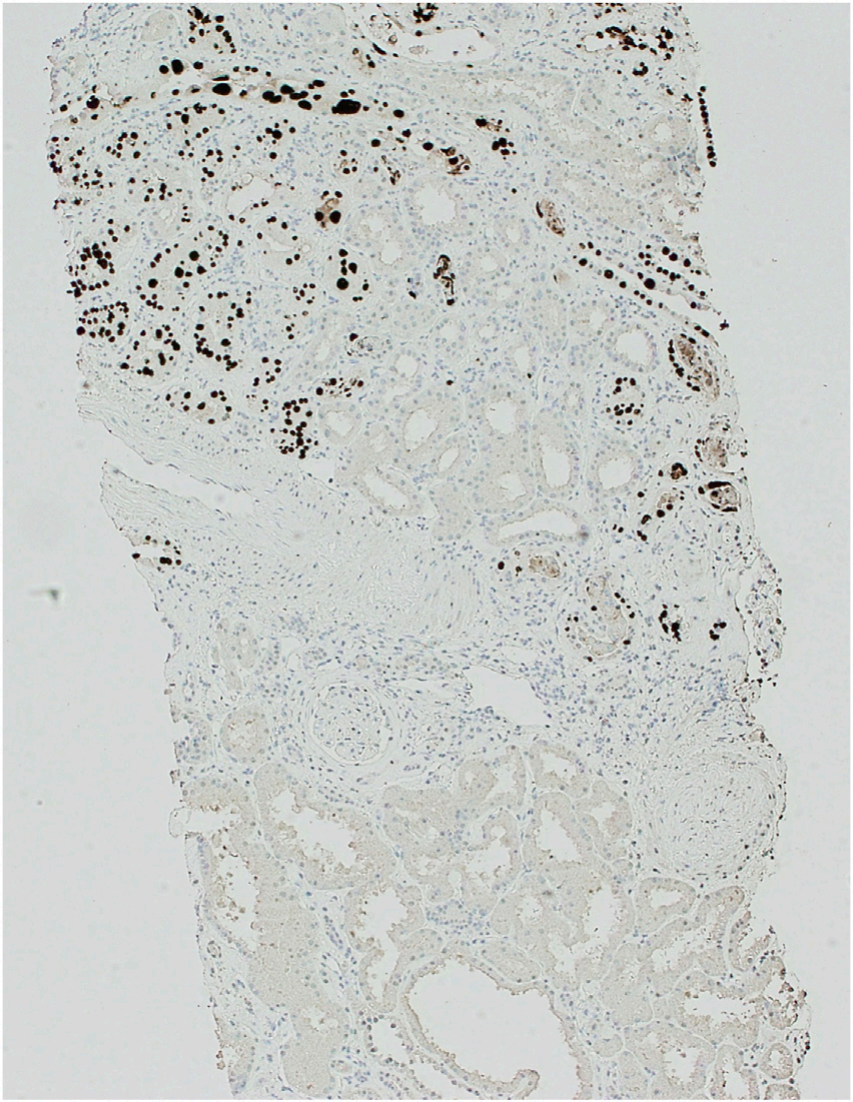
SV40 immunoperoxidase staining of kidney biopsy sample at ×100 magnification. Demonstrating positive (dark brown/black) staining in nuclei of tubular epithelial cells with a patchy distribution.

Over the next year, the patient’s renal transplant function continued to deteriorate, so a further biopsy was performed. This ruled out rejection but revealed severe tubular inflammation with basophilic intra-nuclear inclusions. There was positive SV40 staining in tubular epithelial cells in the areas of inflammation, affecting 10% of tubules (as can be seen in Figure 1). These findings indicated worsening PVAN. The overall appearance was in keeping with Banff PVN Class 2 [9], which is known to be associated with a 25% chance of graft failure after 2 years. Given the previous negative BK virus serology and the repeated positive SV40 staining, this strongly implicated JC-PVAN.

The clinical suspicion of JC-PVAN was confirmed by off-scope PCR in both a biopsy sample Ct 18.74 and a plasma sample Ct 29.07 approximately 6 years post-transplant. The patient’s mycophenolate mofetil was discontinued and tacrolimus was reduced to achieve levels of 4–5 ng/mL. Further plasma samples were sent to the laboratory a month after stopping mycophenolate mofetil, however JC virus was still detectable, Ct 29.19. A follow-up PCR 6 months later remained positive for JC virus, Ct 29.91, with continued renal decline and eGFR at 14 mL/min/1.73 m^2^.

The reduction in immunosuppression allowed for a reduction in the rate of decline in kidney function. However, the patient’s transplant function continued to fall and haemodialysis was restarted 8 years after receiving the kidney transplant.

## Discussion

This case study highlights JC virus-associated nephropathy occurring several years post-transplant. It provides an interesting contribution to the limited knowledge surrounding the unusual presentation of JC-PVAN, which is being increasingly recognised [1, 5, 10–24] and was recently summarised by Gerber et al [24]. It has been hypothesised that, as a result of ongoing graft injury due to drug toxicity, rejection episodes, and donor/recipient human leukocyte antigen mismatch, kidney allograft recipients have the highest risk of developing PVAN compared to other solid organ recipients [25]. Further risk factors for PVAN may include intensity of immunosuppression, immunosuppression with tacrolimus and mycophenolate mofetil, age >50 years, and male gender [13, 15, 24, 26]. Immunosuppression required to prevent allograft rejection may allow the otherwise latent virus to reactivate and cause organ disease [17], however primary JC-PVAN has also been documented [14]. It is important to note that there is variation in the amount of immunosuppressant required between individuals to achieve sufficient suppression of the immune system to prevent transplant rejection whilst reducing risk of viral infections/reactivation. Without a means of determining the degree of immunosuppression achieved, evidence-based standardised regimes are routinely used and, as a result, some patients will initially receive too little immunosuppression and others too much.

To confirm the diagnosis of JC-PVAN, a combination of clinical suspicion, histopathological findings, and virological results are required [18, 27]. Clinical suspicion of BK and JC virus should be high when histopathological findings from biopsy are indicative of PVAN, typically demonstrating inflammation associated with basophilic intranuclear inclusions in tubular and/or Bowman’s epithelial cells [6, 12, 16, 27]. Interestingly, herpes simplex virus, CMV, and adenovirus can cause viral cytopathic changes similar to those of polyomavirus, and therefore SV40 staining is required to confirm the presence of polyomavirus [6, 27]. The infected epithelial cell nuclei are stained with the antibody to the large T antigen of the SV40 virus, which serves as a surrogate marker of human polyomavirus infection [6]. There are limitations to using biopsy for diagnosis, including a risk of sampling uninfected tissue due to the focal nature of early infection and the fact that the SV40 stain cross-reacts with both BK and JC virus [18, 27]. Therefore, PCR is required to help guide diagnosis and confirm species. In this case, PCR was repeatedly negative for BK virus and directed clinical and laboratory teams towards another aetiology.

There are numerous other polyomaviruses that have been identified in humans [28], however, to the best of the authors’ knowledge, the majority are not currently known to cause nephropathy. When there are pathological indications of PVAN and negative BK PCR, clinicians should seek to investigate the presence of JC polyomavirus using techniques such as immunohistochemistry staining with JC-specific antibodies, *in situ* hybridization, or molecular testing of biopsy [18, 27]. Although off scope, the WoSSVC tested a biopsy sample and serially tested plasma, using PCR, to determine JC viremia in this case. In instances of BK nephropathy, our laboratory would also look to test urine to detect active cases, as there is a strong correlation between viremia/viruria and invasive disease [18, 27]. This may be a future consideration for JC virus. Although JC viremia is exclusive to those with PVAN, JC levels may be lower in plasma than urine during infection and plasma may not be a reliable marker [5, 27]. In previous case reports of PVAN, JC virus has been quantified in both urine and plasma [12, 14, 18]. However, if urine is solely used, this may be of limited benefit, with JC found in the urine of both the immunocompetent and immunocompromised. Studies have demonstrated that JC virus is detected in 50%–70% of adults and JC is shed into urine sporadically in approximately 20% of healthy individuals [29–31]. Furthermore, JC has been detected in the urine of asymptomatic patients post kidney transplant [32–34]. It would, therefore, be challenging to differentiate active infection from intermittent shedding with urine alone, and reduction in viruria is not thought a suitable candidate marker for resolution of viral symptoms [35]. In this case, plasma was an appropriate sample type to help to confirm diagnosis, alongside biopsy PCR and histological findings, with further work required to determine the utility of monitoring viral load for response to JC-PVAN interventions.

While the clinical team diagnosed PVAN on histology in this case, the time to confirm that JC was the causative polyomavirus was prolonged. The reason for this was twofold: firstly, repeated BK testing was relied upon to rule out newly acquired BK virus; secondly, testing for JC virus in plasma was initially declined at WoSSVC as it is off scope. Due to the high throughput of clinical samples in our laboratory (>500,000 per year), staff utilise a standardised sample acceptance criteria, with this sample not meeting the inclusion criteria. However, following the second biopsy, the JC PCR requests and clinical information provided from the patient-facing clinical team allowed the samples to be accepted and tested off scope for JC virus. JC was detected in both biopsy and plasma samples.

Although earlier definitive identification of JC virus may not have altered the outcome in this case, it highlights that service improvements could be made with regards to rare presentations of viral infections. This includes promotion of good communication links between patient-facing and laboratory clinical teams via participation in multidisciplinary meetings for complex clinical cases. The WoSSVC will also continue to disseminate information on rare cases, testing complexities and other virological considerations at local and national-level meetings, providing education to a broad range of medical professionals. These actions will help ensure samples from patients with rare diseases are highlighted so that appropriate testing, including those tests out of scope, can be performed on the relevant samples and not delayed. This in turn will allow correct interpretation of results, particularly when tests are off scope. Further, in cases where JC virus is identified, this is beneficial for clinical management outside of PVAN. Those positive for JC virus, when immunosuppressed, are at risk of developing other manifestations such as progressive multifocal leukoencephalopathy [36] and, therefore, detection of JC virus may elevate the index of suspicion.

There are no standardised treatments with regards to JC-PVAN. The Second International Consensus Guidelines on the management of BK Polyomavirus in Kidney Transplantation recommends the reduction of immunosuppression in cases of BK virus viremia in polyomavirus-associated nephropathy [27]. This is to improve immune function to regain control over viral replication, however, it does carry a risk of allograft rejection [37]. Reduction of immunosuppression to stabilise kidney function has also largely been utilised in cases of JC-PVAN [1, 5, 10, 11, 13, 14, 17, 23, 24]. Additionally, there are some examples of using treatments such as intravenous immunoglobulins, cidofovir, leflunomide, or fusidic acid alongside reduction of immunosuppression for BK- and JC-PVAN. However, due to lack of controlled studies it is hard to determine the benefit over reduction of immunosuppression alone [1, 12, 15, 16, 18, 20, 21, 37–39]. In this case, due to the diagnosis of PVAN following SV40 staining, the patient was appropriately managed with stepwise reduction in immunosuppression, alongside monitoring of viremia via Ct values. Unfortunately, this was insufficient to prevent graft failure. Similarly poor outcomes have been detailed in other case studies [1, 10, 13, 14, 18, 20, 24], whilst others demonstrated stabilisation of graft function but no significant improvement of function to pre-JC-PVAN levels [1, 5, 11, 12, 15, 16].

### CONCLUSION

This case highlights that, although rare, JC virus should be considered in the differential diagnosis of nephropathy in immunosuppressed kidney allograft recipients. This is particularly true when biopsy histopathology results demonstrate features in keeping with PVAN and further SV40 staining is indicative of polyomaviruses. PCR should be performed to aid diagnosis, rule out BK virus, and confirm the presence of JC virus. Appropriate awareness of JC virus-associated nephropathy is important for both clinical and laboratory teams. Clinical, histological, and PCR results should be analysed in collaboration between clinical and laboratory teams to ensure appropriate testing and interpretation of results to ensure timely and accurate diagnosis of this rare virus. This work represents an advance in biomedical science because it directly contributes to the currently limited knowledge base on JC-PVAN.

## Data Availability

The original contributions presented in the study are included in the article, further inquiries can be directed to the corresponding author.
